# Electrophysiological Correlates of Object Location and Object Identity Processing in Spatial Scenes

**DOI:** 10.1371/journal.pone.0041180

**Published:** 2012-07-18

**Authors:** Anne H. van Hoogmoed, Danielle van den Brink, Gabriele Janzen

**Affiliations:** 1 Behavioural Science Institute, Radboud University Nijmegen, Nijmegen, The Netherlands; 2 Donders Institute for Brain, Cognition, and Behaviour, Radboud University Nijmegen, Nijmegen, The Netherlands; University of Leuven, Belgium

## Abstract

The ability to quickly detect changes in our surroundings has been crucial to human adaption and survival. In everyday life we often need to identify whether an object is new and if an object has changed its location. In the current event-related potential (ERP) study we investigated the electrophysiological correlates and the time course in detecting different types of changes of an objecṫs location and identity. In a delayed match-to-sample task participants had to indicate whether two consecutive scenes containing a road, a house, and two objects, were either the same or different. In six randomly intermixed conditions the second scene was identical, one of the objects had changed its identity, one of the objects had changed its location, or the objects had switched locations. The results reveal different time courses for the processing of identity and location changes in spatial scenes. Whereas location changes elicited a posterior N2 effect, indicating early mismatch detection, followed by a P3 effect reflecting post-perceptual processing, identity changes elicited an anterior N3 effect, which was delayed and functionally distinct from the N2 effect found for the location changes. The condition in which two objects switched position elicited a late ERP effect, reflected by a P3 effect similar to that obtained for the location changes. In sum, this study is the first to cohesively show different time courses for the processing of location changes, identity changes, and object switches in spatial scenes, which manifest themselves in different electrophysiological correlates.

## Introduction

Ungerleider and Mishkin’s theory [1] proposing that the ventral stream is more active for processing object identity, while the dorsal stream is more active for processing spatial information has been confirmed by many functional magnetic resonance imaging (fMRI) studies and electrophysiological studies in macaques [2]–[8]. In contrast, other studies both in macaques and humans question the validity of a strict dorsal/ventral dual-stream model, showing both streams are active in both identity and location processing [9]–[12]. However, several event-related potential (ERP) studies have revealed different time courses for the processing of object location and object identity information, with some, but not all favoring spatially based information over identity based information [13]–[15]. Furthermore, multiple different ERP correlates have been suggested to be related to the processing of object location and object identity, including N2 and P3 effects [13], [14], [16]–[19]. The posterior N2 effect has been found to be related to the detection of a change [20]–[22], and in a review article by Koivisto and Revonsuo [23] has been interpreted as an instance of the visual awareness negativity (VAN). This VAN is related to the moment an individual becomes aware of a change with respect to visual information held in memory [23], [24]. Posterior N2 effects are often followed by a P3 effect, which has been shown to be related to confidence of the response [21] and to conscious postperceptual processing [23], [24]. On the other hand, the N2 effect has also been found to be followed by a second sustained lateralized negativity when objects have to be encoded in greater detail [25].

All of the neuropsychological studies mentioned above have focused on visual object identification and location processing on blank backgrounds. However, there is reason to believe that objects are processed differently when presented in an environment [26], [27]. Murphy et al. [28] performed an ERP study on locating and identifying objects in a spatial environment, and found that changes in object identity elicited earlier P3 effects than changes in object location. However, in their study, a stronger emphasis was placed on the identity of the objects compared to the location of the objects. After studying the stimuli, during the test phase, participants had to indicate whether the identity of the object was either the same or different as the studied object, regardless of whether the object was in a different location compared to the study phase. In the present ERP study we investigated the time course of detecting different types of changes of an objecṫs location and identity when objects were presented in an environment. We adopted a paradigm in which equal emphasis was placed on both object location and object identity. In addition, we investigated the possible segregation of these kinds of change detections in terms of electrophysiological correlates. Furthermore, we were also interested in the electrophysiological signature of the processing of instances where two objects switch location, in which both spatial and featural information need to be integrated for the detection of a change.

**Figure 1:**
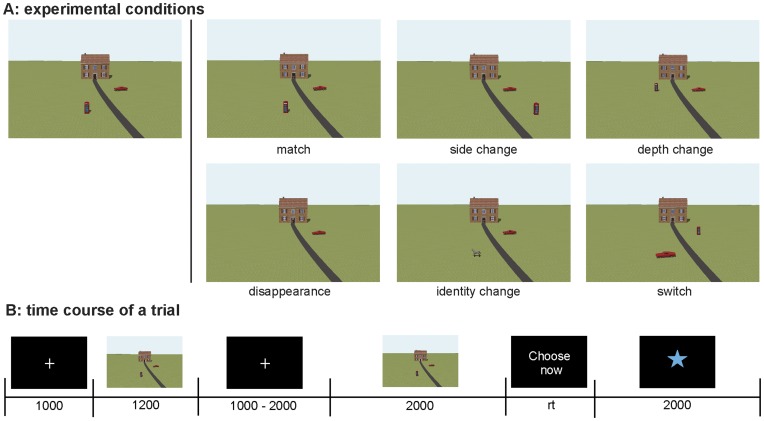
Experimental setup. **A**) Illustrative examples of experimental conditions, **B**) Graphic display of time course of the trials.

To unravel the time course and ERP components related to detecting changes in object identities and object locations in an environment, we presented objects in contextually rich spatial scenes containing a house, a road, and two objects. In order to equally emphasize object identity and object location, we contrasted them directly in a single task in which, using a delayed response paradigm, participants had to decide whether a second scene was identical to a first scene or not. Six conditions were randomly presented in this task (Figure 1A). In (a) the match condition, the second scene was identical to the first scene, in (b) the side change condition, one object’s location changed sides in the visual display, in (c) the depth change condition, one object’s location was moved in depth, in (d) the disappearance condition, one of the objects disappeared, in (e) the identity change condition, one of the objects was replaced by another object, and in (f) the switch condition, the objects switched position, measuring object-to-location binding. Conditions (b), (c), and (d) were all considered location change conditions, since one of the objects left its initial location, while in conditions (e) and (f) both initial locations remained occupied by an object.

Our hypothesis regarding the time course of detecting location and identity changes was that location changes would be processed earlier than identity changes [13], [14]. This is in line with a recent neuronal model on scene processing which proposes that global scene layout is processed first to form a hypothesis on target location, after which this location is processed more deeply to identify the object [29], [30]. Both location changes and identity changes were hypothesized to elicit earlier effects than the object switch condition, since information about the objects identities needs to be bound to their respective locations in order to detect a change and attention to both objects is needed to do so [31]. We expected time course differences between conditions to manifest themselves at the level of the N2 component, reflecting visual change detection processes [20]–[22], and the P3 component, reflecting post perceptual processing [23], [24]. Furthermore, we hypothesized that object location and object identity processing in scenes would manifest themselves in different ERP components [2]–[8], [13], [19].

## Methods

### Ethics Statement

All participants provided written informed consent in accordance with the declaration of Helsinki. This study was approved by the local ethics committee (Commissie Mensgebonden Onderzoek region Arnhem-Nijmegen, The Netherlands).

### Participants

Twenty-three paid volunteers participated in this study, after having given written informed consent. Three participants were excluded from the analyses, two due to problems with the recording computer, and one because of a large number of errors (deviating more than 3 sd from the mean). Thus, twenty participants (10 men, 10 women) remained in the sample. They were all right-handed as assessed by a self-reporting questionnaire. Their mean age was 22.7 years (ranging from 18.2 to 28.5) and they were mostly undergraduate students from the Radboud University Nijmegen. Participants had normal or corrected-to-normal vision and were not color blind.

### Stimuli

The stimuli consisted of computer-generated environments created with Google Sketchup™ (see Figure 1A). This program allows for the creation of 3D environments. These environments are scaled environments and one can measure ‘real life’ sizes of objects and distances between objects in the environment. For example, the front wall of the house measured 6 by 10.5 m. Two objects were placed along the road, which in half of the trials ran from the left bottom of the scene, and in the other half from the right bottom of the scene up to the house centered just above the middle of the scene. The objects could be placed at 4 locations along the road. These locations were either 20 or 38 meters in front of the house (in real size) and were placed at 5 meters horizontal distance from the road (measured from the middle of the object) at both sides. All objects were familiar objects scaled to their normal size. The locations of the objects were counterbalanced. The viewpoint was 72.83 meters from the house, with a viewing angle of 36.6 degrees (10.75 m high) centered at the middle of the front door of the house.

In total, 45 different objects were used to create the stimuli. The object names and real sizes of the objects are reported in Table S1. They were paired differently in each trial, to construct a total of 360 trials. In addition, 4 different objects were used to construct four practice trials. The experiment contained six conditions: a) match, b) side change, c) depth change, d) disappearance, e) identity change, or f) switch (see Figure 1A). In the match condition, the second scene (S2) was the same as the first scene (S1). In the side change condition, one of the objects was moved to the other side of the road in S2, but stayed at the original distance from the house (either 20 or 38 m). In the depth change condition, one of the objects was moved to a new position along the road (from 20 to 38 m or vice versa), but stayed at the same side of the road. In the disappearance condition, one of the objects disappeared. In the identity change condition, one of the objects was replaced by another object. Finally, in the switch condition, the objects from S1 switched positions with each other. For illustration purposes, all scenes in Figure 1A were derived from the same sample scene, but in the experiment no scene appeared in more than one trial. Side change, depth change, and disappearance are all location change conditions, i.e., one of the locations occupied by an object in S1 is not occupied in S2. The scenes were sized 16.7 by 10.6 cm and displayed on a 27×34 cm screen to keep eye-movements as limited as possible. The remaining part of the screen was black.

### Procedure

Participants were equipped with an electrode cap and seated in an electrically shielded room in front of a computer screen at a distance of approximately 60 cm. After they were comfortably seated, the task instruction was visually displayed on the screen. Participants were told that they would be shown two consecutive scenes and they had to press a mouse button after the second picture disappeared to indicate whether the scenes were the same (left button) or different (right button). They were instructed to blink during the presentation of a blinking star displayed in between trials and to blink as little as possible during the rest of the trial. They were also instructed to keep their gaze fixed on the middle of the screen (where the fixation cross was presented), but that they could move their eyes if it was otherwise impossible to see the whole scene.

The time course of a trial is shown in Figure 1B. First, a fixation cross was displayed for 1000 ms, followed by presentation of S1 for 1200 milliseconds. After that a fixation cross was shown which was jittered in duration between 1000 and 2000 milliseconds. Then S2 was presented for 2000 milliseconds followed by the words ‘Kies nu’ (‘Choose now’) which indicated that the participant should respond. After the participant had responded, a blinking star was displayed for 2000 ms, indicating that the participants were allowed to blink. All conditions apart from the match condition contained 45 trials. In the latter condition 135 trials were presented to avoid a bias towards pressing the ‘different’ button.

Four practice trials were used to familiarize the participants with the experimental setting, after which they could ask questions to the experimenter. Next, the experiment started when the participant pressed a button. In total, 5 blocks of 72 trials each were presented to the participant. Each block lasted about 10 minutes and there were breaks in between the blocks.

### EEG Recordings and Analyses

**Figure 2:**
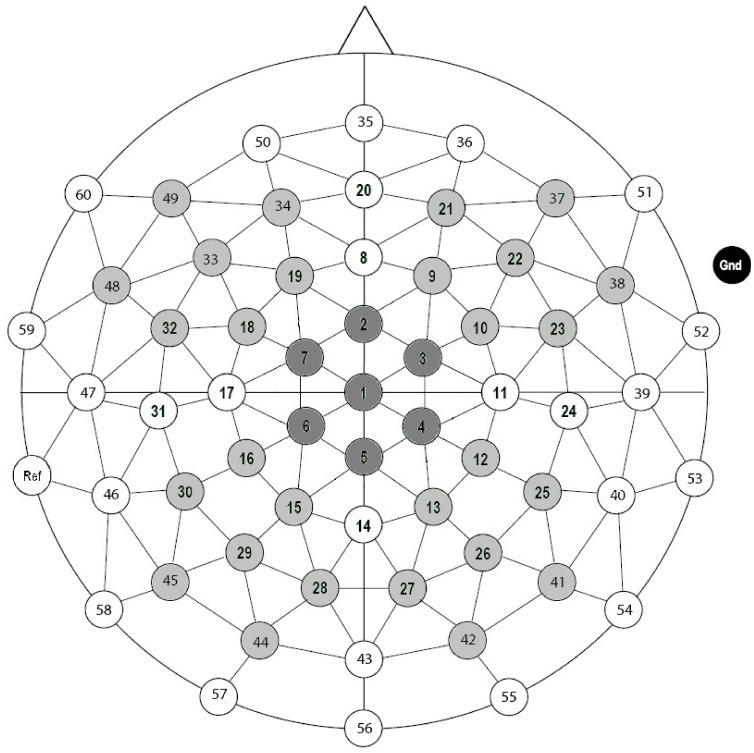
Distribution of electrodes across the scalp with the five clusters selected for statistical analyses.

EEG data were recorded with a 64-electrode equidistant actiCAP (Brain Products GmbH) referenced to the left mastoid (Figure 2). Signals were passed through a BrainAmp DC amplifier (Brain Products GmbH) and were recorded on-line with a sampling rate of 1000 Hz. Measured activity was filtered on-line using a 200 Hz low-pass filter, and a time constant of 10 sec. To measure eye movements, an additional electrode was placed below the left eye. Impedance was kept below 20 KΩ, which is a standard setting in active electrode recording. Data were analyzed in the Matlab-based open source program Fieldtrip [32]. EEG signals were re-referenced to the mean of the left and right mastoid. The signals were screened manually for movement and muscle artifacts and then corrected for horizontal and vertical eye movements by employing the Independent Component Analysis (ICA) method [33]. Signals were filtered with a 0.1 to 40 Hz band-pass filter. Then EEG data were segmented from 200 ms before to 1200 ms after the onset of S2. Segments were baseline-corrected by subtracting the mean amplitude in the −200 to 0 ms pre-stimulus interval. Only correct trials were analyzed. For each electrode ERPs were computed by averaging the segments of artifact-free trials per condition. A mean of 40 to 42 trials per condition were available for the computation of the ERPs with a minimum of 32 epochs per condition per subject. In the match condition, only every third trial was included in the final analysis to keep the number of trials in each condition similar. Mean amplitudes of four latency windows surrounding the peak latency of distinct ERP components were entered into our analyses: Based on our hypotheses, analyses were conducted in latency windows surrounding the N2 (190–250 ms), and P3 (300–500 ms) peaks. In addition, based on visual inspection, mean amplitudes in N1 (80–140 ms) and N3 (250–400 ms) latency windows were analyzed.

Data were analyzed with repeated measures ANOVAs on the mean amplitude values with the within-subject factors Condition (6 levels) and Region (5 levels). For the factor Region, five clusters of seven electrodes were constructed; central (electrodes 1–7), left anterior (18, 19, 32, 33, 34, 48, 49), right anterior (9, 10, 21, 22, 23, 37, 38), left posterior (15, 16, 28, 29, 30, 44, 45), and right posterior (12, 13, 25, 26, 27, 41, 42) (see Figure 2). Greenhousse-Geisser correction for nonsphericity [34] was applied whenever appropriate. Corrected *p* values are reported along with original degrees of freedom.

## Results

### Behavioral Performance

Participants’ performances were highly accurate. Percentages of correctly answered trials per condition are reported in Table 1. Reaction times were not analyzed because participants were instructed to give a delayed response 2 seconds after the stimuli were presented.

**Table 1:** Accuracy Data per Condition.

condition	percentage correct (sd)
match	95.22 (4.49)
side change	98.78 (1.83)
depth change	97.89 (2.93)
disappearance	99.33 (1.62)
identity change	93.33 (5.81)
switch	95.33 (4.14)

### Event-related Potentials

**Figure 3:**
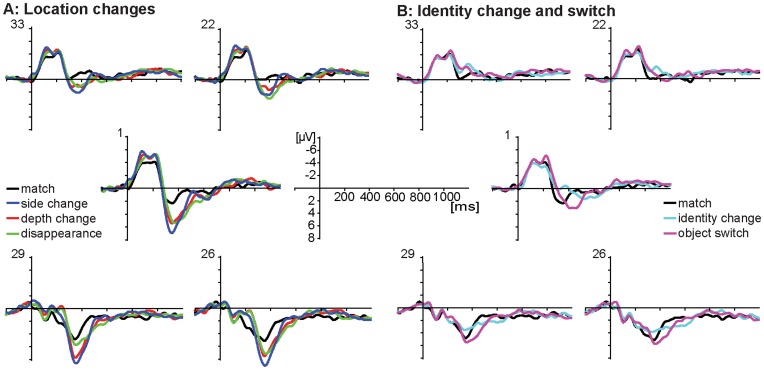
Grand average ERP waveforms of A) location changes, and B) identity changes and switch, both including the match.

The ERP waveforms for all conditions are shown in Figures 3A and 3B. The figures show that the waveforms of the side change, depth change, and disappearance conditions are all deviating from the match around 200 ms after stimulus onset (Figure 3A), while the waveforms for the identity change and switch conditions deviate from the match later at around 300 ms after stimulus onset (Figure 3B). However, the identity change and switch do not resemble each other, with the identity change condition deviating negatively from the match condition and the switch condition revealing a somewhat delayed positive effect compared to the match condition.

#### Omnibus analyses

As a first step, omnibus ANOVAs with the within-subject factors Condition (6 levels) and Region (5 levels) were carried out on the preselected time windows. The statistical results are shown in Table 2. In the N1 latency window (80–140 ms), a fronto-central negativity was found in all conditions, which did not differ between conditions. In the N2 latency window (190–250 ms), a fronto-central negativity was elicited, together with a posteriorly distributed positivity. The ANOVA did not reveal a main effect of Condition, but a significant interaction between Condition and Region was obtained. In the N3 latency window (250–400 ms), a negativity can be seen for the identity change condition compared to the match condition, in the absence of a negativity in the other conditions. This was confirmed by a main effect of both Condition and Region, and an interaction between these factors. In the P3 latency window, Figure 3 illustrates a positive component at central and posterior electrodes in all conditions. The ANOVA revealed a main effect of Condition, a main effect of Region, and an interaction between these two factors. Next, separate ANOVAs were carried out in the N2 and P3 time windows, comparing all conditions separately to the match condition. Whenever a significant effect of Condition or an interaction between Condition and Region was found, differences between conditions were tested separately for each region. Results of these analyses are reported in Table 3 and 4. In the N3 time window an ANOVA was carried out, comparing the identity change to the match, since only the identity change showed a negative effect compared to the match.

**Table 2:** Analysis of variance for the mean ERP amplitudes in the N1, N2, N3 and P3 window for all conditions.

	Condition (*df* = 5,95)		Region (*df* = 4,76)		Condition* Region (*df* = 80,320)	
	*F*	*p*	*F*	*p*	*F*	*p*
N1	.16	.931	59.88	<.001***	1.26	.282
N2	.60	.702	69.62	<.001***	7.65	<.001***
N3	27.78	<.001***	63.77	<.001***	14.80	<.001***
P3	37.27	<.001***	64.51	<.001***	14.06	<.001***

*Note.* *p<.05,

** *p*<.01,

*** *p*<.001.

**Table 3:** Analysis of variance for the mean ERP amplitudes in the N2 window for the non-match conditions compared to the match.

		Side change		Depth change		Disappearance		Identity change		Switch	
	df	*F*	*p*	*F*	*p*	*F*	*p*	*F*	*p*	*F*	*p*
Condition	1,19	.23	.639	.65	.470	.54	.470	.01	.928	.18	.673
Cond* Region	4,76	4.35	.011^*^	13.34	<.001***	9.31	<.001***	.22	.838	.49	.647
Left anterior	1,19	.91	.352	6.05	.024^*^	1.87	.188				
Right anterior	1,19	3.00	.099	.82	.377	1.03	.322				
Central	1,19	.83	.375	.02	.893	.12	.731				
Left posterior	1,19	.21	.631	6.78	.017^*^	8.03	.011^*^				
Right posterior	1,19	1.26	.277	11.83	.003**	15.19	.001**				

*Note.* *p<.05,

** *p*<.01,

*** *p*<.001.

**Table 4:** Analysis of variance for the mean ERP amplitudes in the P3 window for the non-match conditions compared to the match.

		Side change		Depth change		Disappearance		Identity change		Switch	
	df	*F*	*p*	*F*	*p*	*F*	*p*	*F*	*p*	*F*	*p*
Condition	1,19	50.08	<.001***	104.62	<.001***	46.65	<.001***	0.31	.583	18.38	<.001***
Cond* Region	4,76	12.95	<.001***	15.24	<.001***	10.86	<.001***	9.27	<.001***	12.41	<.001***
Left anterior	1,19	23.97	<.001***	67.11	<.001***	36.93	<.001***	5.44	.031^*^	2.98	.100
Right anterior	1,19	42.32	<.001***	71.29	<.001***	40.76	<.001***	3.95	.061	5.45	.031^*^
Central	1,19	50.68	<.001***	119.04	<.001***	56.42	<.001***	.52	.479	21.59	<.001***
Left posterior	1,19	47.49	<.001***	55.57	<.001***	16.27	.001**	1.18	.292	26.06	<.001***
Right posterior	1,19	50.70	<.001***	62.42	<.001***	30.14	<.001***	2.82	.109	32.54	<.001***

*Note.* *p<.05,

** *p*<.01,

*** *p*<.001.

#### N2 latency window

**Figure 4:**
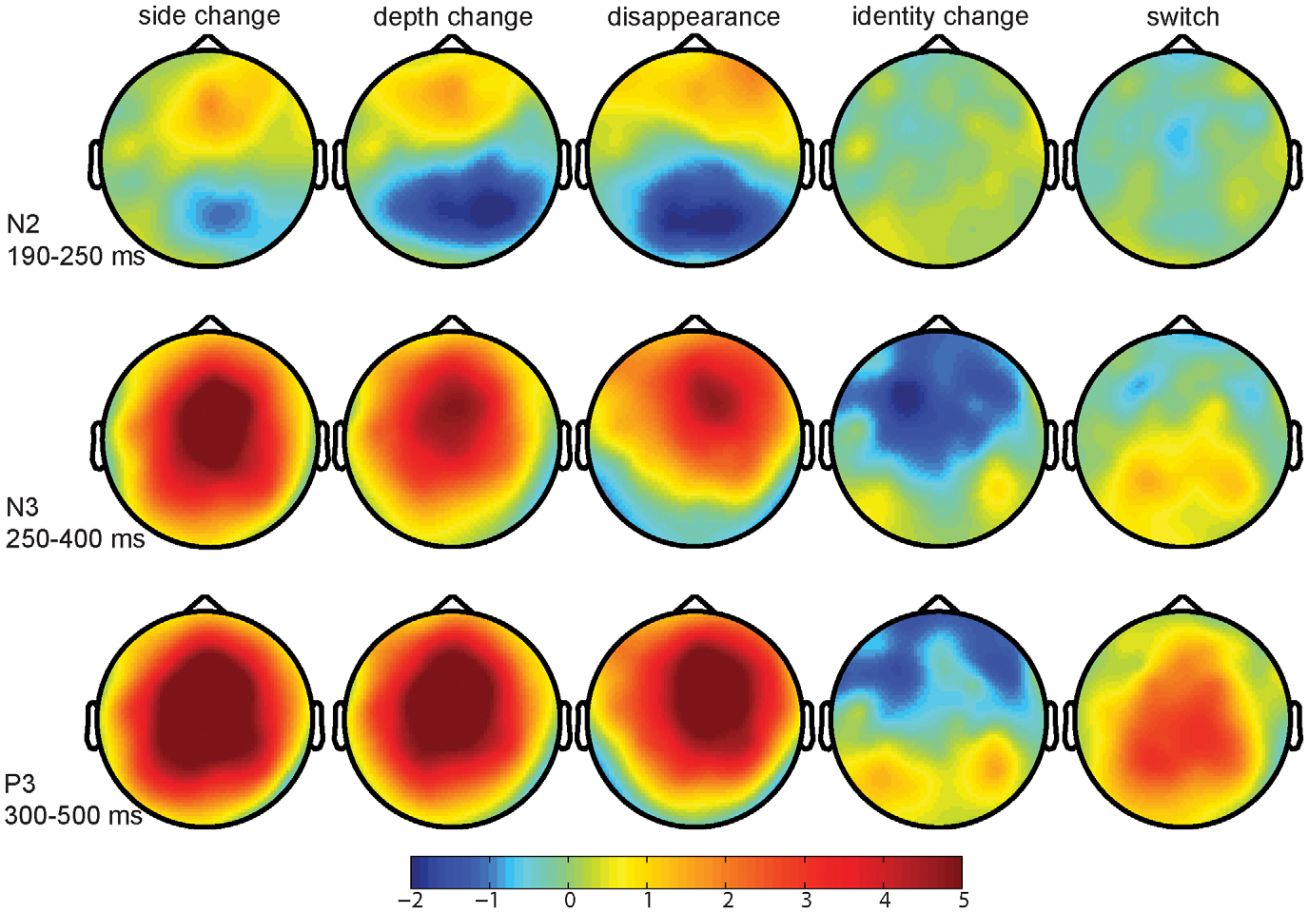
Scalp distributions of ERP effects (change minus match) for N2, N3, and P3 latency windows.

Scalp distributions of all non-matching conditions minus the match condition are shown in Figure 4. Statistical results are reported in Table 3. While the analyses on the side change, depth change, and disappearance conditions all elicited an interaction between Condition and Region, the identity change and switch conditions neither resulted in an effect of Condition, nor in an interaction between Condition and Region. Figure 4 shows that the depth change, and disappearance elicited an anterior P2 and posterior N2 compared to the match condition in this time window, while the identity change and switch conditions did not differ from the match, which was confirmed by the post-hoc analyses. For the side change condition, the interaction was also significant and a similar pattern as in the depth change and disappearance conditions can be observed. However, here post-hoc tests failed to reach significance.

#### N3 latency window

In the N3 latency window, we only observed a negativity in the identity change condition compared to the match condition. Statistical analysis revealed a marginally significant effect of Condition (*F* (1,19) = 3.61, *p = *.073), a main effect of Region (*F* (4,76) = 80.73, *p*<.001), and an interaction between these factors (*F* (4,76) = 5.93, *p = *.002). Identity change was found to elicit an anterior N3 effect compared to the match (LA: *F* (1,19) = 8.92, *p* = .008, RA: *F* (1,19) = 7.13, *p* = .015).

#### P3 latency window

Statistical results of the ANOVAs in the P3 window are reported in Table 4. Scalp distributions of differences between the non-matching conditions and the match condition are shown in Figure 4. The results revealed a significant main effect of Condition for all contrasts involving the match condition except for the identity change versus match. In addition, significant interactions between Condition and Region were found for all comparisons against the match. For the side change, depth change, and disappearance conditions the P3 effect was significant in all regions, with a maximum over the central areas (see Figure 4). In the switch condition, the P3 effect had a less widespread distribution across the scalp, confirmed by significant main effects in all but the left anterior region. In the identity change condition, significant effects of condition were only found in the anterior regions. However, as can be seen in Figure 4, this is due to a negativity of the identity change compared to the match instead of a P3 effect. However, in a slightly later time window, the identity change seems to show a P3 effect compared to the match condition. Additional statistical analyses of the 450–650 ms latency window did reveal a significantly larger amplitude in the identity change condition than the match condition at central and posterior sites (all *p*<.005).

#### Side change, depth change, and disappearance

We were interested in differences between the different location change conditions. A difference between side and depth change versus disappearance would indicate that the scenes are processed further after one detects that the object has disappeared from its original location. A difference between side change and depth change could indicate a difference between categorical and coordinate changes [35], [36].

The three location change conditions were tested against each other in the N2 and P3 time windows. In the N2 window no effect of Condition and no interaction between Condition and Region were obtained. In the P3 time window, no main effect of Condition, but a significant effect of Region (*F* (4,76) = 52.71, *p*<.001), and an interaction between Condition and Region (*F* (8,152) = 6.51, *p*<.001) were found. Side change elicited a larger P3 effect than depth change in the right posterior region (*F* (1,19) = 5.83, *p = *.026). The P3 in the disappearance condition was more right lateralized than in the side change and depth change conditions. This was confirmed by a larger positivity at the left posterior sites for both side and depth change compared to disappearance (*F* (1,19) = 21.64, *p*<.001, and *F* (1,19) = 13.01, *p* = .002, respectively).

#### Identity change vs. switch

The identity change and switch were tested in a Condition (2)×Region (5) repeated measures ANOVA in the N2, N3 and P3 latency windows. In the N2 latency window, no effect of Condition, and no interaction between Condition and Region were found. In the N3 latency window, effects of Condition (*F* (1,19) = 11.26, *p* = .003) and Region (*F* (4,76) = 76.10, *p*<.001) were obtained, but no interaction between the two factors. Identity change elicited a larger anterior negativity and a smaller posterior positivity than the switch. In the P3 latency window effects of Condition (*F* (1,19) = 31.91, *p*<.001), as well as Region (*F* (4,76) = 74.76, *p*<.001), and an interaction between Condition and Region (*F* (4,76) = 5.74, *p* = .005) were found. Switch elicited a larger positivity in all regions (all *F*s>13.5, *p*≤.002). This effect was larger in the central region than in the other regions (all *F*s>9.72, *p*≤.006), and it was smaller in the right posterior region relative to the left posterior region (*F* (4,76) = 5.42, *p* = .031).

## Discussion

In the present ERP study, we investigated the time course and electrophysiological correlates of processing object location and object identity in spatial scenes. In a delayed match-to-sample task participants had to indicate whether two consecutive scenes, both containing a road, a house, and two objects, were either the same or different. The scenes were identical, one of the objects had changed identity, one of the objects had changed location, or the objects had switched positions. The results show that location changes are detected earlier than identity changes, which in turn are detected faster than object switches. These effects are reflected by modulations of the N2, N3, and P3 components.

Relative to the match, all location change conditions revealed a posterior N2 effect in the 190–250 ms latency window, although in the side change condition this effect did not reach statistical significance (see Figure 4). The observed N2 effect reflects the detection of a change in a visual stimulus and could be interpreted as an instance of the VAN [21]–[24]. These results show that in this early time window, location changes have already been detected, while identity changes and object switches have not. Importantly, the effect in this time window cannot be explained by differences in visual attention between the conditions, as the conditions were randomly intermixed in this study. Moreover, it has been shown that differences in attention to visual stimuli are reflected in an N1 effect [37], and we did not find an effect of condition in the N1 window. Therefore, we interpret the posterior N2 effects as reflecting the detection of a visual change.

The results show that ERPs to identity changes deviate somewhat later from the match condition than location changes, resulting in an N3 effect in the 250–400 ms latency window. One may argue that this negative effect reflects a delayed VAN, as it has previously been shown that the latency of the VAN can be delayed until 460 ms depending on the contrast between the stimuli [23]. However, the N3 effect in our study reaches its maximum amplitude at anterior electrode sites whereas the VAN has been shown to have a posterior scalp distribution. These differences in scalp distribution indicate that the N3 effect is functionally different from the N2 effect in the location change conditions. Indeed, a similar anteriorly distributed N3 effect has previously been found to reflect processing of object specific representations [20], [38]. Only after objects presented in the scene have been identified and compared to object representations in memory, a difference can be detected. These results show that object specific processing within an environment takes place within 400 ms after presentation of the second stimulus.

The 300-to-500 ms time window revealed P3 effects for several conditions. Amplitude modulations of the P3 component were found for location changes and the switch compared to the match and identity change. The effect was even larger for the location changes compared to the switch. These results are in line with previous studies presenting objects on blank screens showing larger P3 amplitudes for location changes relative to matches [16], and a larger amplitude for location changes than identity changes [17]. The switch condition did not deviate from the match until this time window, suggesting that the switch was detected only after 300–500 ms, and thus later than the location change and identity change. This is in line with the theory that both information about location and identity of the object have to be bound together, and this requires integration of information processed in the dorsal and ventral stream [1].

The only condition that did not show a larger P3 compared to the match in the 300 to 500 ms latency range, was the identity change condition. However, the identity change did elicit a small P3 effect in a slightly later time window. This later occurrence of the positive effect in the identity change condition is possibly due to the elicitation of an overlapping N3 component in the identity change condition. Alternatively, the apparent P3 latency difference between the identity change condition and the location change conditions may also reflect a longer duration of perceptual and decision-related processing in the identity change condition [21], [28], [39], [40]. This would be an indication that the detection of the identity changes requires more effort than the detection of location changes. In addition, irrespective of the latency difference, the P3 amplitude of the identity change appears to be smaller than the amplitude of the location changes. The P3 amplitude has previously been shown to be inversely related to the difficulty of stimulus evaluation [39], [40] and to the confidence of the response [21], [41], providing further evidence for more effortful processing of identity changes relative to location changes.

The present study also provided an opportunity to test for more subtle differences between multiple types of location changes. We, therefore, compared the side change, depth change, and disappearance condition with each other. Results show that only the P3 component was modulated differently in these conditions. An effect was shown for side change and depth change compared to disappearance. While the P3 was centrally distributed for the side and depth change, in the disappearance condition it was more right lateralized. This implies that scenes in which an object has moved to another place are processed differently from a scene in which one of the objects has disappeared. Moreover, it shows that despite being able to perform the task while only attending to the locations that were previously filled with an object, participants processed the whole scene.

Side change and depth change also slightly differed in P3 effect size, with side change eliciting a larger P3 effect than depth change only in the right posterior region. This might be due to a difference between categorical and coordinate processing, which has been shown to modulate P3 amplitude [35], and in the present study would be reflected in the side versus depth change. On the other hand, participants may have perceived both the side and the depth change as a categorical change, since there were only two possible depths which could be coded as near and far. Alternatively, the difference in P3 amplitude may be due to the relative difficulty of detecting a depth change compared to the side change. Whereas in the depth change condition the object changed visual appearance, i.e. it changed size, in the side change condition the visual appearance remained the similar, making change detection in the depth change condition harder [40], [42]. Also, the side change condition resulted in a larger difference in the retinal image compared to the depth change condition. Future research controlling for the degree of change in the retinal image may elucidate the nature of this small P3 effect.

When comparing identity change to the switch, we found an N3 effect, similar to the one obtained in the identity change to match comparison. Also, we found a larger P3 amplitude for the switch compared to the identity change. These findings imply that two objects switching position is not merely processed as two object changing identity at two distinct locations, but can be considered a functionally distinct category of change detection.

In sum, our results show that when objects are presented within a spatial scene, the time courses of detecting location changes, identity changes, and object switches differ from each other. Location changes were detected already within 250 ms after presentation of the second stimulus as shown by a modulation of a posteriorly distributed N2 component, followed by a P3 effect. Detection of identity changes occurred later, but within 400 ms and elicited an anterior N3 effect, followed by a delayed and reduced P3 effect. In contrast, object switches were detected within 500 ms as reflected in the modulation of the P3 only, resembling the P3 effect found in location changes. These differences in neural correlates of the detection of location changes and detection of identity changes is in line with results showing that these types of information are processed in different visual streams [2]–[8]. The ERP time courses of the different contrasts suggest that location changes are easiest to detect followed by identity changes and finally object switches.

It may be argued that these findings are inherent to the processing of the scenes in our paradigm. If one is aware that possible changes always involve the objects in the scene, one will direct attention to the two locations that previously contained an object. A location change can then be detected directly by noticing the object has disappeared, whereas for detecting an identity change, the object first needs to be processed further in terms of object representations. For the detection of an object switch, spatial and featural information first need to be combined, causing a delay in the detection of these changes. Alternatively, the observation of time course differences for location and identity processing may translate to ecologically valid environments, where location changes of objects in the immediate environment (e.g. an object approaching) are detected before the object’s identity. This has been proposed in two visual search models and confirmed by behavioral, MEG and effective connectivity results, showing that low spatial frequency components of a scene are processed first, guiding spatial attention to an object that is processed subsequently [27], [28], [43]–[45]. However, in order to strengthen the claim that location change is processed before identity change, replication of a temporal advantage of location over identity in different spatial environments would be beneficial.

To conclude, human beings are able to detect any changes in an objecṫs location, object’s identity, and switches of objects extremely fast, even when these objects are embedded in a context. Our results suggest that location changes are detected faster than identity changes. To appropriately act and function in our environment is crucial to human adaptation and survival. The fast processing of changes in our surroundings are the first steps of a human neural mechanism that allows adjustment of behavior. Being aware of changes in an objecṫs location may indeed be more important for accurate adaption of behavior than changes of an objects identity. For example, fast detection and processing of the locations and changes in location of objects such as vehicles are important in everyday life when crossing the street. This study is the first to cohesively show that the human neural system is fine-tuned to change detection and moreover differentiates between different types of changes in an environment.

## Supporting Information

Table S1 **Pictures of the objects used in constructing the stimuli and their size in the virtual 3D environment.**
(DOCX)

## References

[1] Underleider LG, Mishkin M (1982). Two cortical visual systems. In Ingle MA, Goodale MI, Masfield RJW, editors. Analysis of Visual Behavior. Cambridge, MA: MIT Press.. Pp.

[2] Duhamel J-R, Bremmer F, BenHamed S, Graff W (1997). Spatial invariance of visual receptive fields in parietal cortex neurons.. Nature.

[3] Haxby JV, Horwitz B, Ungerleider LG, Maisog JM, Pietrini P (1994). The functional organization of human extrastriate cortex: a PET-rCBF study of selective attention to faces and locations.. J Neurosci.

[4] Jackson MC, Morgan HM, Shapiro KL, Mohr H, Linden DE (2011). Strategic resource allocation in the human brain supports cognitive coordination of object and spatial working memory.. Hum Brain Mapp.

[5] Munk MHJ, Linden DEJ, Muckli L, Lanfermann H, Zanella FE (2002). Distributed cortical systems in visual short-term memory revealed by event-related functional magnetic resonance imaging.. Cereb Cortex.

[6] Pihlajamäki M, Tanila H, Könönen M, Hänninen T, Aronen HJ (2005). Distinct and overlapping fMRI activation networks for processing of novel identities and locations of objects.. Eur J Neurosci.

[7] Tanaka K, Saito H, Fukada Y, Moriya M (1991). Coding visual images of objects in the inferotemporal cortex of the macaque monkey.. J Neurophysiol.

[8] Ungeleider LG, Haxby JV (1994). ‘What’ and ‘where’ in the human brain.. Curr opin neurobiol.

[9] Cichy RM, Chen Y, Haynes J-D (2011). Encoding the identity and location of objects in human LOC.. NeuroImage.

[10] Jellema T, Maassen G, Perrett DI (2004). Single cell integration of animate form, motion and location in the superior temporal cortex of the macaque monkey.. Cereb cortex.

[11] Op de Beeck H, Vogels R (2000). Spatial sensitivity of macaque inferior temporal neurons.. J Comp Neurol.

[12] Sereno AB, Maunsell JHR (1998). Shape selectivity in primate lateral intraparietal cortex.. Nature.

[13] Singhal A (2006). Differentiating between spatial and object-based working memory using complex stimuli: an ERP study.. Int J Neurosci.

[14] Mecklinger A, Pfeifer E (1996). Event-related potentials reveal topographical and temporal distinct neuronal activation patterns for spatial and object working memory.. Cognitive Brain Res.

[15] Wu C-T, Libertus ME, Meyerhoff KL, Woldorff MG (2011). The temporal dynamics of object processing in visual cortex during the transition from distributed to focused spatial attention.. J Cogn Neurosci.

[16] Simon-Thomas ER, Brodsky K, Willing C, Sinha R, Knight RT (2003). Distributed neural activity during object, spatial and integrated processing in humans.. Cognitive Brain Res.

[17] Mecklinger A (1998). On the modularity of recognition memory for object form and spatial location: a topographic ERP analysis.. Neuropsychologia.

[18] Mecklinger A, Meinshausen R-M (1998). Recognition memory for object form and object location: an event-related potential study.. Mem Cognition.

[19] Mecklinger A, Müller N (1996). Dissociations in processing of ‘what’ and ‘where’ information in working memory: an event-related potential analysis.. J Cogn Neurosci.

[20] Eddy M, Schmid A, Holcomb PJ (2006). Masked repetition priming and event-related brain potentials: A new approach for tracking the time-course of object perception.. Psychophysiology.

[21] Eimer M, Mazza V (2005). Electrophysiological correlates of change detection.. Psychophysiology.

[22] Potts GF, Liotti M, Tucker DM, Posner MI (1996). Frontal and inferior temporal cortical activity in visual target detection: Evidence from high spatially sampled event-related potentials.. Brain Topogr.

[23] Koivisto M, Revonsuo A (2010). Event-related brain potential correlates of visual awareness.. Neurosci Biobehav R.

[24] Koivisto M, Revonsuo A (2003). An ERP study of change detection, change blindness, and visual awareness.. Psychophysiology.

[25] Bornstein MH, Mash C, Aterberry ME (2011). Young infants’ eye-movements over “natural” scenes and “experimental” scenes.. Infant behav dev.

[26] Tsivilis D, Otten LJ, Rugg MD (2001). Context effects on the neural correlates of recognition memory: an electrophysiological study.. Neuron.

[27] Mazza V, Caramazza A (2011). Temporal brain dynamics of multiple object processing: the flexibility of individuation.. PlosOne.

[28] Murphy JS, Wynne CE, O’Rourke EM, Commins S, Roche RAP (2009). High-resolution ERP mapping of cortical activation related to implicit object-location memory.. Biol Psychol.

[29] De Cesarei A, Loftus GR (2011). Global and local vision in natural scene identification.. Psychon Bull Review.

[30] Huang T-S, Grossberg S (2010). Dynamics of contextually cued attentive visual learning and search: spatial and object evidence accumulation.. Psychol rev.

[31] MacEvoy SP, Epstein RA (2011). Constructing scenes from objects in human occipitotemporal cortex.. Nat neurosci.

[32] Oostenveld R, Fries P, Maris E, Schoffelen JM (2011). FieldTrip: Open Source Software for Advanced Analysis of MEG, EEG, and Invasive Electrophysiological Data.. Comp Intelligence and Neurosci.

[33] Jung T-P, Makeig S, Humphries C, Lee T-W, McKeown MJ (2000). Removing electroencephalographic artifacts by blind source separation.. Psychophysiology.

[34] Greenhouse SW, Geisser S (1959). On methods in the analysis of profile data.. Psychometrika.

[35] Van der Lubbe RHJ, Schölvinck ML, Kenemans JL, Postma A (2006). Divergence of coordinate and categorical spatial processing assessed with ERPs.. Neuropsychologia.

[36] Van der Ham IJM, van Strien JW, Oleksiak A, Van Wezel RJA, Postma A (2010). Spatial working memory for categorical and coordinate information: an ERP study.. Int J Psychophysiol.

[37] Johannes S, Münte TF, Heinze HJ, Mangun GR (1995). Luminance and spatial attention effects on early visual processing.. Cognitive Brain Res.

[38] McPherson WB, Holcomb PJ (1999). An electrophysiological investigation of semantic priming with pictures of real objects.. Psychophysiology.

[39] Czigler I, Csibra G (1990). Event-related potentials in a visual discrimination task: negative waves related to detection and attention.. Psychophysiology.

[40] Hagen GF, Gatherwright JR, Lopez BA, Polich J (2006). P3a from visual stimuli: task difficulty effects.. Int J Psychophysiol.

[41] Busch NA, Fründ I, Herrmann CS (2010). Electrophysiological evidence for different types of change detection and change blindness.. J Cognitive Neurosci.

[42] Kok A (2001). On the utility of P3 amplitude as a measure of processing capacity.. Psychophysiology.

[43] Bar M, Kassam KS, Ghuman AS, Boshyan J, Schmid AM (2006). Top-down facilitation of visual recognition.. PNAS.

[44] Kveraga K, Boshyan J, Bar M (2007). Magnocellular projections as the trigger of top-down facilitation in recognition.. J Neurosci.

[45] Treisman AM, Gelade G (1980). A feature-integration theory of attention.. Cognitive Psychology.

