# Supplementary figures and images for: Electrophysiological Correlates of Object Location and Object Identity Processing in Spatial Scenes
Source: PLoS One. 2012 Jul 18;7(7):e41180. doi: 10.1371/journal.pone.0041180 (PMC3399828; doi:10.1371/journal.pone.0041180)

| *Object* | *Height (cm)* | *Width (cm)* | *Object* | *Height (cm)* | *Width (cm)* |
| --- | --- | --- | --- | --- | --- |
| 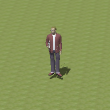 | 180 | 50 | 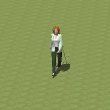 | 178 | 54 |
| 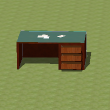 | 125 | 235 | 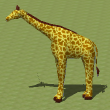 | 345 | 385 |
| 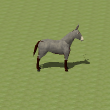 | 163 | 183 | 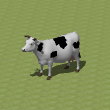 | 157 | 252 |
| 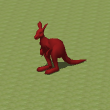 | 184 | 268 | 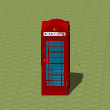 | 221 | 80 |
| 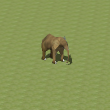 | 97 | 136 | 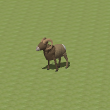 | 127 | 133 |
| 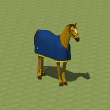 | 230 | 240 | 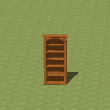 | 182 | 92 |
| 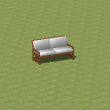 | 140 | 78 | 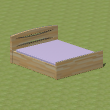 | 268 | 109 |
| 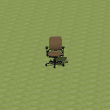 | 96 | 68 | 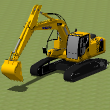 | 338 | 624 |
| 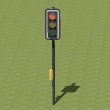 | 351 | 55 | 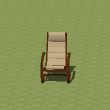 | 156 | 66 |
| 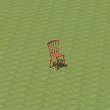 | 95 | 58 | 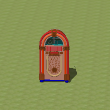 | 198 | 113 |
| 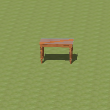 | 126 | 78 | 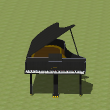 | 250 | 219 |
| 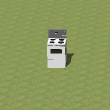 | 117 | 62 | 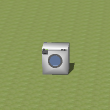 | 101 | 98 |
| 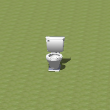 | 118 | 72 | 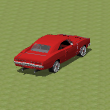 | 104 | 373 / 130 |
| 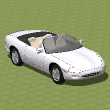 | 138 | 477 | 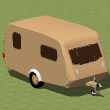 | 210 | 476 |
| 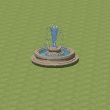 | 148 | 180 | 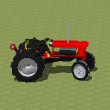 | 213 | 293 |
| 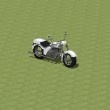 | 115 | 233 | 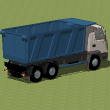 | 248 | 563 |
| 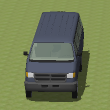 | 200 | 579 | 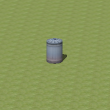 | 81 | 55 |
| 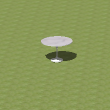 | 66 | 108 | 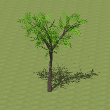 | 300 | 255 |
| 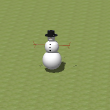 | 148 | 145 | 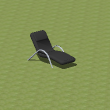 | 109 | 191 |
| 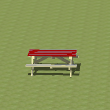 | 84 | 190 | 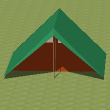 | 207 | 381 |
| 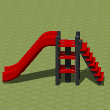 | 190 | 384 | 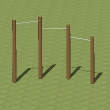 | 241 | 305 |
| 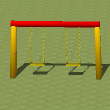 | 214 | 343 | 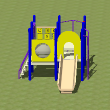 | 203 | 266 |
| 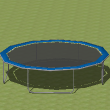 | 93 | 432 |  |  |  |
|  |  |  |  |  |  |

Supplement: Table S1 — Pictures of the objects used in constructing the stimuli and their size in the virtual 3D environment. (DOCX) [file pone.0041180.s001.docx]
